# Current Studies of Mitochondrial Quality Control in the Preeclampsia

**DOI:** 10.3389/fcvm.2022.836111

**Published:** 2022-02-28

**Authors:** Xiaoqing Peng, Ruirui Hou, Yuanyuan Yang, Zhigang Luo, Yunxia Cao

**Affiliations:** ^1^Department of Obstetrics and Gynecology, The First Affiliated Hospital of Anhui Medical University, Hefei, China; ^2^National Health Commission Key Laboratory of Study on Abnormal Gametes and Reproductive Tract (Anhui Medical University), Hefei, China; ^3^School of Pharmacy, Anhui Medical University, Hefei, China; ^4^The Key Laboratory of Major Autoimmune Diseases, Anhui Medical University, Hefei, China; ^5^Department of Cardiovascular, The First Affiliated Hospital of Anhui Medical University, Hefei, China

**Keywords:** mitochondrial quality control, mitophagy, biogenesis, fusion, fission, preeclampsia

## Abstract

Mitochondria are cellular energy powerhouses that play important roles in regulating cellular processes. Mitochondrial quality control (mQC), including mitochondrial biogenesis, mitophagy, mitochondrial fusion and fission, maintains physiological demand and adapts to changed conditions. mQC has been widely investigated in neurodegeneration, cardiovascular disease and cancer because of the high demand for ATP in these diseases. Although placental implantation and fetal growth similarly require a large amount of energy, the investigation of mQC in placental-originated preeclampsia (PE) is limited. We elucidate mitochondrial morphology and function in different pregnancy stages, outline the role of mQC in cellular homeostasis and PE and summarize the current findings of mQC-related PE studies. This review also provides suggestions on the future investigation of mQC in PE, which will lead to the development of new prevention and therapy strategies for PE.

## Preeclampsia

Preeclampsia (PE) is a leading cause of neonatal and maternal morbidity and mortality, affecting 2–8% of pregnant women worldwide (1, 2). Preeclampsia is diagnosed by new-onset hypertension (systolic>140 mmHg and diastolic>90 mmHg) after 20 weeks of gestation accompanied by one or more other features: proteinuria, other maternal organ dysfunction (including liver, kidney and neurological), hematological involvement, and/or uteroplacental dysfunction, such as fetal growth restriction and/or abnormal Doppler ultrasound findings of utero-placental blood flow (3). Pre-term delivery is often the only definite treatment for PE, which is associated with adverse short- and long-term health outcomes in offspring, including a high prevalence of subsequent endocrine and metabolic diseases in children (4). Other effective treatment options are limited.

**Figure 1:**
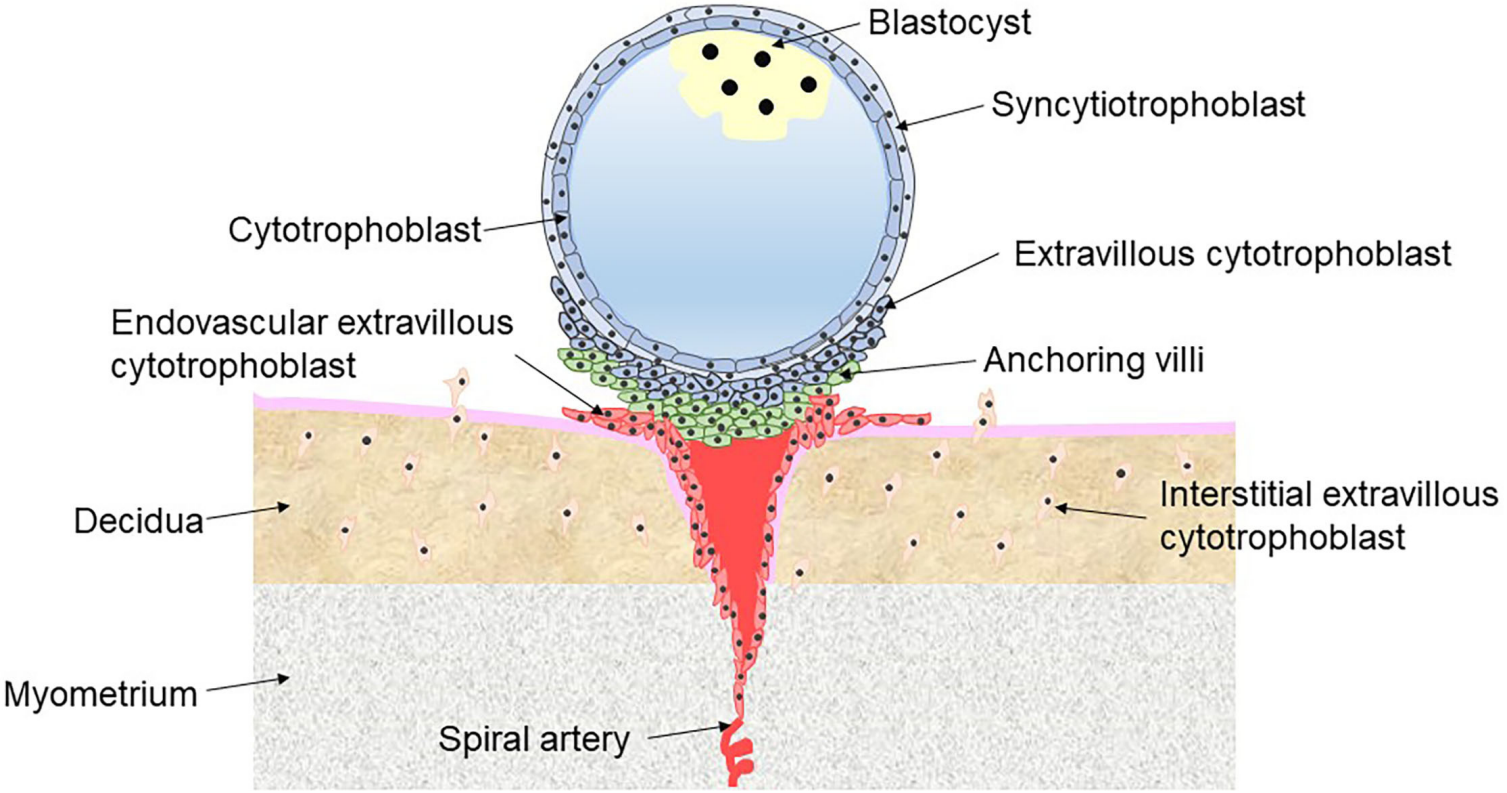
Different types of trophoblast in placental implantation.

PE is a placental interface-originated disease affecting multiple organ systems (5). Abundant evidence suggests that defective implantation of placentation is the core risk factor for PE, characterized by abnormal trophoblast invasion and remodeling of the spiral arteries (6). Under normal conditions, the blastocyst is encapsulated by a shell of cytotrophoblast (CT) cells, which adhere to the uterus, penetrate into the decidua and continue to proliferate and differentiate in the first trimester. CT is an undifferentiated and proliferative trophoblast that either fuses into multinucleated syncytiotrophoblast (ST) on the surface of the shell or differentiates into extravillous cytotrophoblast (EVT) through a partial epithelial-mesenchymal transition at the interface between the outer surface of the shell and the tips of anchoring villi. ST cells facilitate the uptake of nutrients and oxygen from maternal blood and produce large quantities of placental hormones (including progesterone and hCG) to maintain pregnancy. There are two types of EVT. Interstitial EVT migrates into the lumen of the maternal decidua via the invasion of the endometrium, while endovascular EVT invades the myometrial spiral arteries involving the remodeling spiral arteries (Figure 1) (7). The myometrial spiral arteries are remodeled from high-resistance, coiled vessels to dilated low-resistance vessels because of the intervillous space at the terminal portion entered by endovascular EVT. Remodeling of the myometrial spiral arteries adapts to the increased cardiac output during pregnancy and slows the blood flow into the intervillous space of placenta, meeting the oxygen and nutrition requirements of the developing fetus (8).

Shallow placental implantation and defective spiral artery remodeling lead to placental ischemia, releasing angiogenic markers such as soluble fms-like tyrosine kinase 1 (sFlt-1) and soluble endoglin (sEng) (9). Impaired placental implantation could be triggered by the abnormalities of CT fusion into ST or abnormalities of ST differentiation into EVT (10). Flt-1 is a receptor of vascular endothelial growth factor (VEGF) and placental growth factor (PIGF), which are mediators of the transformation from epithelial to endothelial phenotype to regulate the endothelial cell function. sFlt-1, a splice variant of Flt1 lacking the trans-membrane and cytoplasmic domains, performs as an antagonist of VEGF and PIGF resulting in endothelial dysfunction (11). Similarly, sEng is the shed Eng from the endothelial cell surface into maternal circulation, which binds to transforming growth factor beta 1 in circulation. Thus, free transforming growth factor beta 1 is decreased, and the migration and proliferation of endothelial cells are inhibited (9). Elevated sFlt-1 and sEng have been found in PE in dozens of human studies (9) and thus have been recognized as predictive or diagnostic biomarkers of PE (12, 13). These antiangiogenic factors lead to the following vasoconstrictive state, oxidative stress and microemboli that contribute to the clinical features of PE (9). Moreover, the administration of sEng or sFlt-1 has been shown to induce severe PE signs or adverse birth outcomes in pregnant rats (11, 14).

## Mitochondrial Morphology and Function During Pregnancy

Mitochondria are the main resource of energy production for placental implantation and development. Adenosine triphosphate (ATP) synthesis requires five subunit protein complexes (i.e., complexes I-V) of the electron transport chain through oxidative phosphorylation in the inner membrane of the mitochondrion (IMM). Nicotinamide adenine dinucleotide (NADH) and flavin adenine dinucleotide 2 (FADH2) produced from the tricarboxylic acid cycle expedite electrons to the electron transport chain at complex I (NADH dehydrogenase) and complex III (ubiquinone cytochrome c reductase), respectively. The electrons flow to Complex IV, reducing O_2_ to H_2_O. Meanwhile, protons (H^+^) are transferred from the mitochondrial matrix to the intermembrane space at complexes I, III, and IV, leading to the proton gradient and transmembrane electrical potential. The energy stored at proton gradient is used to synthesize ATP (15).

The mitochondrion is a double-membrane organelle with an ion-permeable IMM and an outer mitochondrial membrane (OMM) (16). Mitochondrial morphology and function vary in different trimesters of pregnancy. In the first trimester, CT differentiation into ST leads to a shift from the classical morphology of mitochondria (0.2–0.8 μm) with lamellar cristae into small (<0.1 μm), irregular shapes with no defined cristae and low-density matrix, and this adaptation meets the increased requirement of energy production in mitochondria for embryo development (17). The smaller mitochondria in ST might facilitate the transport of cholesterol and steroidogenesis, which requires cytochrome P450scc and 3 s-hydroxysteroiddehydrogenase-Δ^4−5^ isomerase type I located in the IMM, to transform cholesterol into pregnenolone and then convert into progesterone (18). Sufficient steroid hormone progesterone synthesized in human placental mitochondria is essential for the maintenance of pregnancy (19). With the development of the placenta, the mitochondrial content is greater in the third trimester than in the first trimester (20). However, the respiratory rate in the third trimester is similar to that in the first trimester. Thus, the efficiency of mitochondrial respiration using oxygen is lower in the later trimester after normalization to the mitochondrial content (20).

Mitochondria consume 90% cellular O_2_ to synthesize ATP and are thus sensitive to oxygen tension. In the early first trimester (6–10 weeks), the spiral arteries are plugged by endovascular EVT so that oxygen tension is lower around the placenta, ~20 mmHg in the placenta and ~60 mmHg in the decidua (21). Relative hypoxia limits ATP synthesis by mitochondria, but the endometrial glands consume D-glucose to supply a large amount of ATP (22). Low O_2_ pressure promotes trophoblast proliferation and angiogenesis in the placenta (23, 24). With embryo growth, the spiral arteries become unblocked at the end of the first trimester, and the oxygen tension rises to ~60 mm Hg at the placenta and ~70 mm Hg at the decidua through the villous tress to meet the increased metabolic requirement (21). High O_2_ pressure promotes CT fusion into the ST and further invasion in the placenta (23, 25).

Hypoxic condition increases the secretion of sFlt-1 that related to the pathogenesis of PE (26). Hypoxia has been shown to reduce mitochondrial content, mitochondrial oxidative capacity and the expression of key molecules involved in the electron transport chain (27, 28). However, the dynamic alteration of mitochondrial morphology and function in PE has never been observed, which should be investigated in the future to comprehensively understand the role of mitochondria in the pathology of PE.

## Reactive Free Radicals and PE

Reactive free radicals (ROS) are byproducts of oxidative phosphorylation, including superoxide ($O_{2}^{-}$), hydrogen peroxide (H_2_O_2_), hydroxyl radical (OH) and peroxynitrite (ONOO^−^). Most ROS are produced when electrons leak from complexes I/III: the leaked electrons reduce O_2_ to generate $O_{2}^{-}$, of those generated at complex I are delivered into the matrix and of those generated at complex III are released into both the matrix and the intermembrane space, and then the dismutation of $O_{2}^{-}$ to H_2_O_2_ is induced by superoxide dismutase 2 (SOD2) in the matrix and SOD1 in the intermembrane space. Glutathione peroxidases and peroxiredoxins are antioxidant enzymes that decompose H_2_O_2_ to $O_{2}^{-}$. The balance between ROS and antioxidant defense maintains cellular physical function. During pregnancy, a low level of ROS upregulates transcription factor E26 transformation-specific oncogene homolog 1 and VEGF to promote angiogenesis (29) and increases mitogen-activated protein kinase (MAPK) signaling to facilitate trophoblast differentiation and placental development (30).

Excessive ROS overwhelm antioxidant defense, leading to detrimental effects on cell physiologies such as lipids, proteins and DNAs. Excessive ROS production and an impaired enzymatic antioxidant system are detected in PE (31). Both the direct measurement of $O_{2}^{-}$/H_2_O_2_ and the indirect measurement of oxidative phosphorylation capacity (complex I-IV, cytochrome c oxidase) are reduced in PE (32, 33). Moreover, alterations in various proteins involved in oxidative phosphorylation have been found (34, 35). On the other hand, the expression and activity of antioxidant enzymes, including SODs, GPXs, thioredoxin reductases and catalase, are suppressed in PE placentas and trophoblasts (36). The decreased expression and activity of antioxidant enzymes result in the low efficiency of ATP synthesis, leading to electronic leakage and subsequent high production of ROS (37). ROS accumulation then triggers increased lipid peroxidation, including malondialdehyde (MDA), thiobarbituric acid reactive substances (i.e., a production of MDA) and 4-hydroxynonenal-modified proteins (38, 39).

Several well-known antioxidant nutrient supplementations have been found to prevent PE in small randomized trials, but a meta-analysis of randomized controlled trials revealed that vitamin C and E, selenium, L-arginine, allicin, lycopene or coenzyme Q10 did not effectively prevent PE (40). This could be because that antioxidants increase the concentration of circulating antioxidants but cannot repair the imbalance between ROS and antioxidants (41). Moreover, a recent study (42) found that potent antioxidant MitoQ administration during late gestation alleviated PE, but treatment during early gestation exacerbated reduced uterine perfusion pressure (RUPP)-induced PE in mice. Mild ROS has been shown to improve the proliferation, invasion and migration of CT-characterized HTR8-S/Veno cells for early placental implantation, and this could be blunted by antioxidants (42). Because mitochondrion is the main source of ROS, the lack of an effect of antioxidant therapy on PE brings out the consideration whether that mitochondrial-targeted interventions would be effective in preventing PE (40).

## Mitochondrial Quality Control and PE

**Figure 2:**
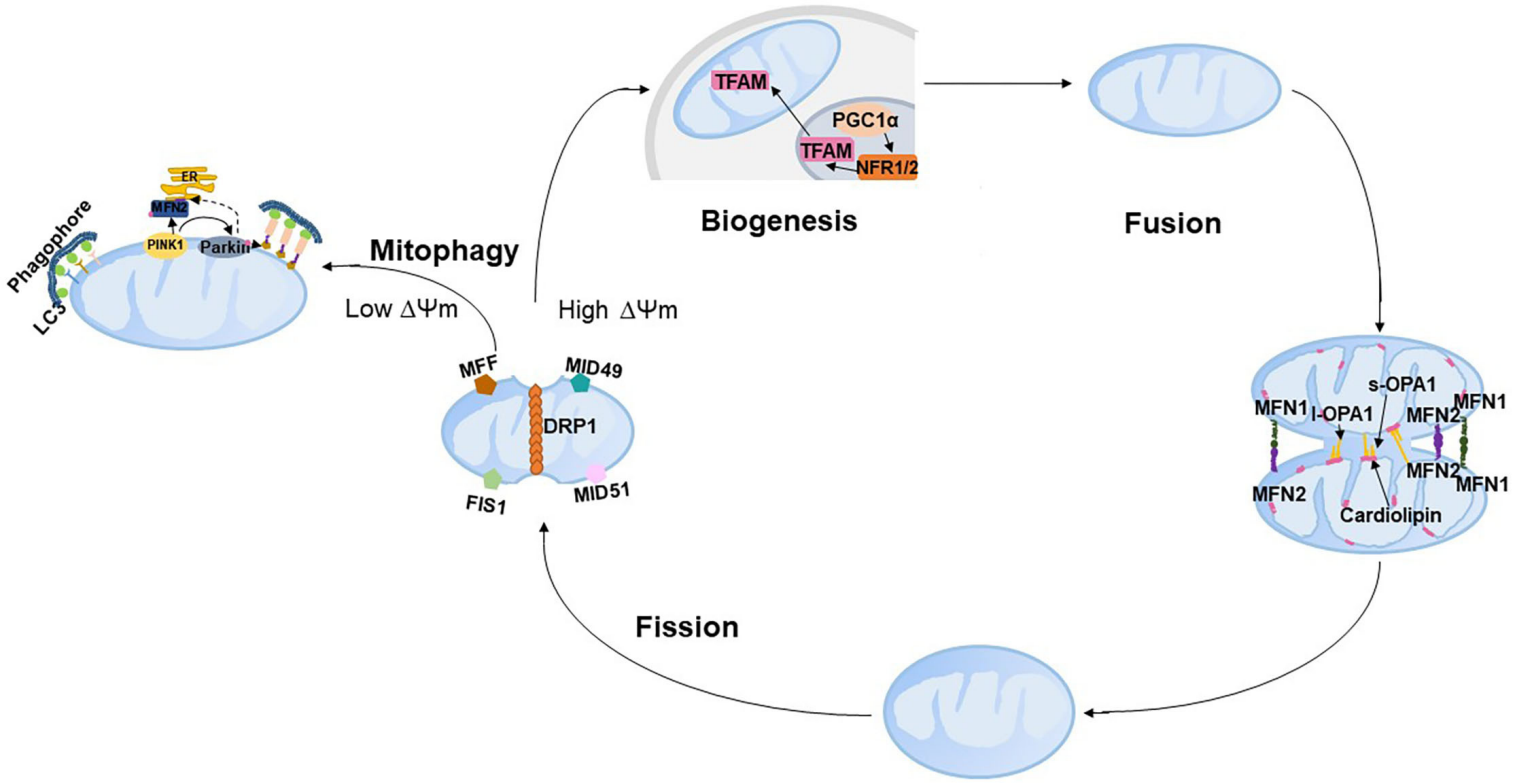
Schematic representation of mitochondrial quality control. PINK, PTEN-induced putative kinase 1; LC3, microtubule-associated protein 1 light chain 3; ER, endoplasmic reticulum; DRP1, dynamin-related protein 1; FIS1, fission 1; MFF, mitochondrial fission factor; MID49, mitochondrial dynamics protein of 49 kDa; and MID51, mitochondrial dynamics protein of 51 kDa.

Mitochondria cannot be synthesized *de novo* but contain their own self-replicating genome. Coordination between mitochondrial autophagy (mitophagy) and biogenesis to deal with irreparably damaged mitochondria is essential for maintaining the mitochondrial volumes and determining the rate of mitochondrial turnover. Damaged mitochondrial proteins or parts of mitochondrial organelles are removed by mitophagy, and damaged components are renewed by adding proteins and lipids through biogenesis. Both mitochondrial biogenesis and mitophagy require mitochondrial dynamics fusion and fission. Mitochondrial dynamics, including mitochondrial fission and fusion nested in the tube-like mitochondrial network, continuously occur in response to metabolic or environmental stresses such as caloric restriction and low temperature. The integration of fusion, fission, mitophagy and mitochondrial biogenesis is referred to as mitochondrial quality control (mQC, Figure 2). The following text will introduce the process of mQC processes and the related predominant proteins. To our knowledge, 10 articles reported the expression of mQC-related genes in PE (Table 1), and this will also be overviewed.

**Table 1:** Summary of current studies in humans related to mitochondrial quality control molecules.

**Articles**	**Groups (*n* and gestation weeks)**	**Parts**	**Sites**	**Alteration**	**Non-alteration**
Wangkheimayum et al. (41)	13 30.5 ± 2.9 wks eoPE, 11 37.8 ± 1.0 wks loPE, 14 39.2 ± 0.9 wks Ctrl	Placenta	1.5–2 cm next to the umbilical cord insertion, 1 cm in depth	mRNA and protein of OPA1 in eoPE	mRNA of MFN1, MFN2, NRF among three groups; mRNA and protein of OPA1 in eoPE; mRNA of TFAM in loPE; Protein of DRP1 among three groups.
				mRNA of TFAM in lope protein of TRAM in eoPE	
Yang et al. (42)	16 36.4 ± 2.26 wks PE, 16 36.7 ± 1.96 wks Ctrl	Villous tissues	NA	mRNA of MFN2	
Ventura-Clapier et al. (43)	10 33.7 ± 1.2 wks eoPE, 10 30.2 ± 1.1 wks loPE, 10 32.7 ± 1.4 wks Ctrl	Myometrial biopsy (0.5 ×0.5 ×0.5 cm)	the upper edge of lower segment uterine incision	Protein of TFAM, PGC-1α in lope; mRNA of OPA in eoPE; Protein of L-OPA1:S-OPA1 in eoPE.	mRNA of NRF1 and NRF2 among three groups; Protein of MFN1, MFN2, DRP1, PINK1 and BNIP3 among three groups
				Protein of s-OPA in both PE	
Brenmoehl and Hoeflich (44)	11 30.0 ± 3.9 wks sPE, 11 31.0 ± 4.3 wks Ctrl,	Placenta	the maternal side of the placental villous tissue	Protein of MFN1, MFN2, OPA1, BNIP3, and PGC-1α	Protein of DRP1 and FIS1
Ryan and Hoogenraad (45)	33 29.3 ± 3.0 wks PE, 30 29.7 ± 2.3 wks Ctrl	Placenta	NA	Protein of DRP; protein of p-DRP1 in MIs	NA
				Protein of OPA1	
Virbasius and Scarpulla (46)	14 37.88 ± 2.10 wks term PE, 20 38.75 ± 0.84 wks term Ctrl, 8 29.73 ± 3.21 wks pre-term PE, 10 29.29 ± 3.83 wks pre-term Ctrl	Placenta	NA	Protein of l-OPA1:s-OPA1 in term PE vs. term Ctrl; Protein of MFN1 in term PE vs. term Ctrl.	Protein of DRP1 in placenta and placental MIs between two term groups and between two pre-term groups; Protein of MFN2 between two term groups and between two pre-term groups.
				Protein of FIS1 in term PE vs. term Ctrl;	
Matsubara et al. (32)	20 32.45 ± 1.81 wks eoPE, 20 38.29 ± 1.60 wks Ctrl,	Placenta	NA	Protein of BNIP and MFN2	NA
Huo and Scarpulla (47)	19 <34 wks pre-term PE, 20 <34 wks pre-term Ctrl	Placenta	NA	mRNA and protein of PGC1α	NA
Ristevski et al. (48)	12 33 ± 3 wks PE, 11 39 ± 1 wks Ctrl	Placenta (<1 cm^2^)	the paracentral region of the placenta at the maternal side	Protein of NRF1, BNIP3, BCL2, BNIP3L; mRNA and protein of DNM1.	mRNA of PGC1α, NRF2α, FUNDC1, FIS1, MFN1, MFN2 and OPA1; Protein of TFAM, FUNDC1, PINK1, PARK2.
				Protein of PGC1α; mRNA of NRF1, TFAM, BNIP3, BCL2, BNIP3L, PINK1, and PARK2	
Li et al. (49)	10 PE, 10 Ctrl	Placenta	NA	Ubiquitination level of FUNDC1	

*wks, weeks; eoPE, early-onset PE; loPE, late-onset PE; Ctrl, normotensive controls; MIs, mitochondrial isolates; the listed molecules with highlight in gray refers increase in those molecules, and the listed molecule highlighted in gray refers decrease in those molecules; the mRNA or protein were in default extracted from placentas unless specified to MIs; and, the alteration was in default compared with controls unless specified otherwise*.

### Mitochondria Biogenesis

Mitochondrial biogenesis produces new mitochondria based on pre-existing mitochondria to respond to internal and external stresses such as oxidative stress, inflammation and mitochondrial drug toxicity. Mitochondrial biogenesis involves synthesis of IMM and OMM and mitochondrial encoded proteins; replication of mitochondrial DNA (mtDNA); and synthesis and import of nuclear encoded mitochondrial proteins. The vast majority of mitochondrial proteins are encoded by the nuclear genome, and thus mitochondrial biogenesis requires exquisite coordination of both mitochondrial and nuclear genomes, such as target, importation and correction of mRNA from nuclear to mitochondria (43). Peroxisome proliferator-activated receptor gamma coactivator 1-alpha (PGC-1α) is the master regulator of mitochondrial biogenesis, which is activated by either phosphorylation or deacetylation in the cytoplasm and then translocates to the nucleus (44, 45). Activated c-1α in the nucleus stimulates the expression of two key transcription factors, nuclear respiratory factor 1 (NRF1) and NRF2, and further interacts with NFR1/2 to increase their transcriptional activity, leading to the increased activity of mitochondrial transcription factor A (TFAM) to replicate mtDNA and encode mitochondrial proteins (46). Moreover, NRF1/2 are essential for nuclear-mitochondrial crosstalk to adapt to mitochondrial biomass and oxidative metabolism, especially in the developmental stage. NRF1-null mice exhibit lethality at early embryos due to the dramatic lack of mtDNA content and mitochondrial membrane potential in blastocysts (47). Embryos homozygous for the null NRF2 allele die prior to implantation, which highlights the critical mitochondrial roles of NRF2 during cleavage events of the embryo (48). PGC-1α also interacts with other nuclear transcription factors, such as estrogen-related receptors, thyroid hormone, peroxisome proliferator-activated receptors, and glucocorticoids, to regulate mitochondrial energy metabolism, respiration, and biogenesis (43).

**Figure 3:**
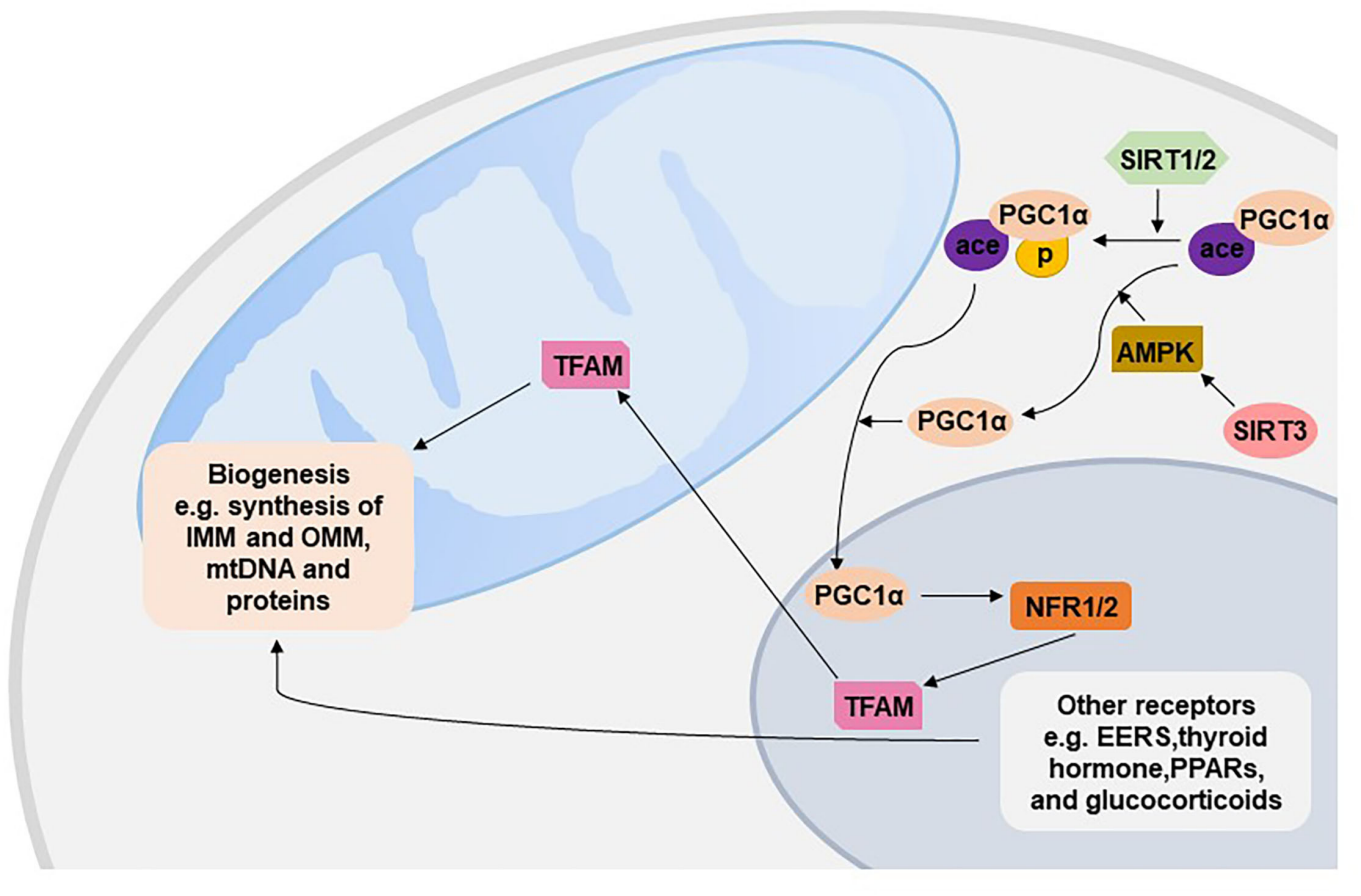
Mitochondrial biogenesis. EERs, estrogen-related receptors; PPARs, peroxisome proliferator-activated receptors; TFAM, mitochondrial transcription factor A; PGC1α, peroxisome proliferator-activated receptor gamma coactivator 1-alpha; NFR, nuclear respiratory factor; SIRT, Sirtuin; AMPK, AMP-activated kinase; IMM, inner membrane of the mitochondrion; OMM, outer membrane of the mitochondrion.

Mitochondrial biogenesis can be regulated by several cell signaling pathways (49). AMP-activated kinase (AMPK) activated by exercise and starvation directly phosphorylates PGC-1a or indirectly deacetylates PGC-1a to stimulate biogenesis (50). The human Sirtuin isoforms SIRT1-2 have been shown to deacetylate PGC-1a and then increase the activity of PGC-1a (51, 52); SIRT3 has been shown to increase the expression of PGC-1a through AMPK (Figure 3) (44). Recently, a decreasing trend of SIRT1 and activated AMPK/PGC-1a protein have been found in PE placentas (53), and inhibited SIRT1 and PGC-1a have been found in a group of patients with both intrauterine growth restriction and PE (54). Moreover, downregulated proteins of both SIRT3 and PGC1a were found in severe PE (55). Numerous regulators, including calcium/calmodulin-dependent protein kinase IV, Akt, AMP-dependent protein kinase (PKA), NO, and PPARa, also activate mitochondrial biogenesis (56).

Recent studies have shown decreased protein levels of PGC-1α in PE patients compared with controls (53, 55, 57), and the transcriptional level of PGC-1α was reduced in pre-term PE and pre-term controls (53) but was not altered between pre-term PE relative to term counterparts (57). All the current studies only assessed the mRNA or protein level of PGC-1α; however, activated PGC-1α (i.e., phosphorylated or deacetylated PGC-1α) has never been reported, and the expression of PGC-1α was not specified in the cytoplasm or mitochondrion. The protein level of NRF1 in pre-term PE patients has been shown to be higher than that in term controls, while the mRNA level of NRF1 was lower (57). Another study reported no difference in either the protein or mRNA expression of NRF1 between pre-term PE and pre-term counterparts (58). The number of cases of each group in the above three studies was relatively similar (i.e., 10–20), and the inconsistent results might result from the unmatched gestational age at delivery and unspecified subcellular organelles (e.g., nucleus or cytoplasm) from which PGC-1α was extracted. NFR2 has only been found to be decreased in hypoxia-induced BeWo cells (27). TFAM was 1.8-fold lower in late-onset PE placentas but not altered in early-onset PE placentas compared with control placentas (59).

### Mitophagy

**Figure 4:**
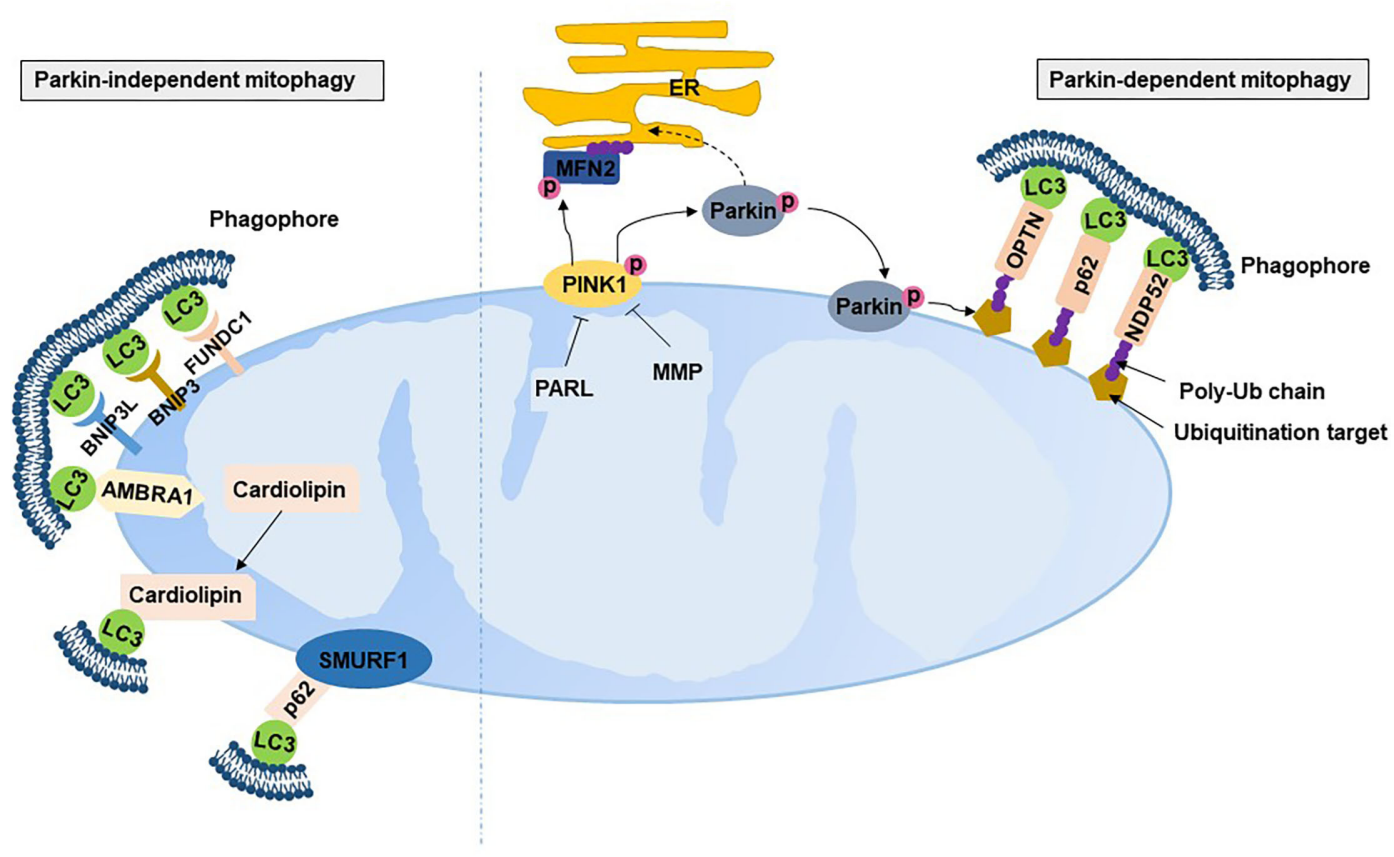
Mitophagy. PARL, presenilin-associated rhomboid-like protease; MMP, mitochondrial-processing protease; PINK, PTEN-induced putative kinase 1; NDP52, nuclear dot protein 52; OPTN, optineurin; LC3, microtubule-associated protein 1 light chain 3; BNIP3, Bcl2/adenovirus E1B 19 kDa protein-interacting protein 3; BNIP3L, Bcl2/adenovirus E1B 19 kDa protein-interacting protein 3-like; FUNDC1, fun14 domain containing 1; AMBRA1, autophagy and beclin 1 regulator 1; SMURF1, SMAD specific E3 ubiquitin protein ligase 1.

Mitophagy is the selective autophagic degradation of damaged mitochondria by autophagosomes and lysosomes. The best studied mitophagic process is the PTEN-induced putative kinase 1 (PINK1)-Parkin mitophagy pathway. PINK1 is a serine/thereonine kinase mainly localized to the OMM (60). Under normal conditions, PINK1 located at the OMM is imported into the IMM through the translocase of the inner and outer membrane complex and then cleaved and degraded by proteases, including mitochondrial-processing protease and inner membrane presenilin-associated rhomboid-like protease (PARL) (61–63). In contrast, damaged mitochondria with an indication of depolarized membrane potential are unable to import and degrade PINK1, resulting in the accumulation of PINK1 on the OMM (61). The accumulated PINK1 on the OMM is activated by autophosphorylation, which phosphorylates the E3 ubiquitin ligase Parkin by phosphorylating Thr175 and Thr217 on Parkin's linker region and translocates Parkin from the cytosol to mitochondria (61). Activated Parkin binds to phosphorylated ubiquitin tethering to the OMM, leading to the conjugation of ubiquitin to various substrates and the formation of polyubiquitin (poly-Ub) chains (64). The poly-Ub chain provokes the recruitment of Ub-binding autophagy receptors, including p62/sequestosome 1, nuclear dot protein 52 and optineurin, to connect with microtubule-associated protein 1 light chain 3 (LC3) and further facilitates the selective engulfment of ubiquitinated mitochondria by the autophagosome (Figure 4). A recent study has revealed that ubiquitination of the mitofusion 2 (MFN2) is one of the very first step of mitophagy that occurs prior to autophagosomal engulfment of the organelle (65). PINK1 can explicitly facilitate Parkin-dependent mitophagy and be activated in other manners. This is evidenced by Parkin mutants having more severe phenotypes than PINK-null flies (66), while Parkin overexpression rescued mitochondrial morphology (67) and arrested mitochondrial motility (68). Parkin also simulates mitochondrial biogenesis, presumably to replace damaged mitochondria with healthy and functional organelles by degrading transcriptional repression (i.e., parkin-interacting substrate) on the depolarized mitochondrion (69).

Mounting evidence suggests that there are Parkin-independent mitophagic mechanisms. Several Parkin-independent proteins localize to mitochondria to recruit autophagosomes by interacting with LC3, including Bcl2/adenovirus E1B 19 kDa protein-interacting protein 3 (BNIP3), BNIP3-like (BNIP3L) and Fun14 domain containing 1 (FUNDC1) (70). BNIP3 and BNIP3L bonding to Bcl-2 separates the complex of Bcl-2 with Beclin-2, resulting in the initiation of autophagosomes (71). BNIP3 and BNIP3L have been shown to be complementary to mitophagy (71) and protect against excessive ROS (72). FUNDC1 localized on the OMM can be dephosphorylated under hypoxia to interact with LC3 on autophagosome membranes (73, 74). In addition, SMAD-specific E3 ubiquitin protein ligase 1 and autophagy and beclin 1 regulator 1 also induce LC3 dependence in a Parkin-independent mitophagic manner (75, 76). Cardiolipin, a membrane lipid in IMM, is also an LC3 receptor. Cardiolipin translates from the IMM to the OMM with external adverse stimulation and then interacts with the N-terminal helices of LC3 (77), indicating that cardiolipin also participates in mitophagy. Studies have found that mitochondria-derived vesicles stimulated by ROS instead of mitochondrial depolarization induced a faster rate of mitochondrial turnover with the requirement of Pink1/Parkin by delivering the mitochondrial content to the lysosome, where degradation of the mitochondrial content occurs (78, 79).

Compared with Parkin-dependent mitophagy, Parkin-independent mitophagy tends to play a more crucial role in PE. One study found that PINK1_63kDa/53kDa_ ratio was increased in line with the increase in Parkin in the placentas of PE (80). PINK1 is 63 kDa under normal conditions, and cleaved PINK1 is 53 kDa. Mitophagy, as indicated by the PINK1 and Parkin proteins, was exhibited in the placentas of PE mice (81). In contrast, BNIP3-mediated mitophagy has been found to be involved in PE, evidenced by the higher protein expression of BNIPB and BNIP3L and higher mRNA expression of FUNDC1 in pre-term PE placentas compared to term controls (57). BNIP3 was inhibited in term severe PE placentas compared with term controls (55), while BNIP3 was upregulated in the pre-term early-onset PE placentas compared with the term controls (34). The ubiquitination level of FUNDC1 was low in hypoxic HTR8-S/Veno cells and the placenta of pregnant women with PE (82).

### Mitochondrial Fusion

**Figure 5:**
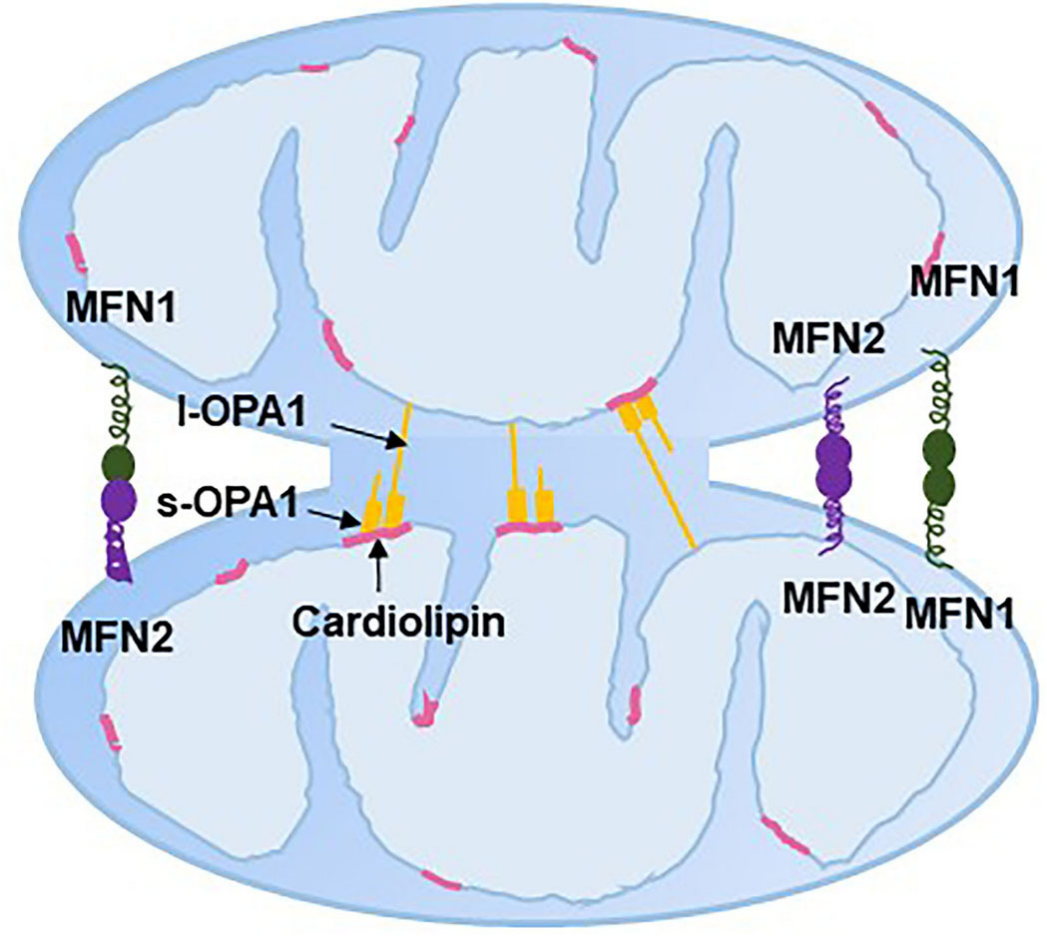
Mitochondrial fusion. ER, endoplasmic reticulum; MFN, mitofusin; l-OPA1, long-optic atrophy 1; s-OPA1, short-optic atrophy 1.

Mitochondrial fusion helps to mitigate metabolic or environmental stresses by distributing the mitochondrial contents between partially damaged mitochondria and healthy mitochondria (83). The fused mitochondria can be prevented from mitophagy (84). Mitochondrial fusion includes the fusion of both OMM and IMM and a mixture of mitochondrial contents. Mitochondrial fusion in mammalian cells is regulated by the fusion proteins MFN1/2 on the OMM and optic atrophy 1 (OPA1) on the IMM, which all belong to the dynamin-related family of large nucleotide guanosine triphosphates (GTPases). The GTPase domain of MFN1/2 hydrolyses GTP, which promotes homo- and hetero-oligomerization of MFN to dock on two OMMs and initiates OMM fusion (85–87) (Figure 5). Although the GTPase activity of MFN1 was ~eightfold higher than that of MFN2, the affinity for GTP of MFN2 was more than 100-fold higher than that of MFN1 (85). This could be because that the internal interaction between the first and second heptad repeat domains of MFN2 resulted in the closed conformation of MFN2 and sequent fusion-deficiency. The closed conformation is activated by the phosphorylation at MFN2 Ser 378 (88). Replacing Ser 378 with Asp that cannot be phosphorylated has normal MFN2-mediated fusion features (88). Moreover, genetic mutations of MFN2 in murine embryonic fibroblasts interrupt mitochondrial fusion and produce large mitochondrial fragments (89). Interestingly, mutation in human MFN2 but not MFN1 results in Charcot–Marie–Tooth disease type 2A, a neurodegenerative disorder disease (90, 91). However, in MFN1-deficient Hela cells, mitochondrion failed to bind to each other and resulted in fragmentation of mitochondrion (92). Loss of either MFN1 or MFN2 causes lethality in mice, and the extracted cells from these mice display obviously fragmented mitochondria (87). These evidences suggest that MFN1/2 are dispensable for mitochondrial fusion.

OPA1 typically has two isoforms: long/membrane-bound (l-OPA1) and short/soluble OPA1 (s-OPA1). l-OPA1 is located in the IMM, and s-OPA1 is integral in the intermembrane space. l-OPA1 can be further cleaved to s-OPA1, while overexpression of s-OPA1 leads to mitochondrial fragmentation, which might be the result of mitochondrial fission (93). The interaction between l-OPA1 on one IMM and cardiolipin on another IMM has been shown to be essential to mitochondrial fusion *in vitro*, with evidence that the absence of cardiolipin caused the loss of membrane fusion activity (94). Whether s-OPA1 is required for IMM fusion is still controversial. Ishihara et al. (95) and Tondera et al. (96) found that l-OPA1 is sufficient to facilitate IMM fusion, while other studies showed that both s-OPA1 and l-OPA1 are required for efficient and fast fusion (97, 98). Moreover, the l-OPA1:s-OPA1 ratio is thought to mediate the balance between fission and fusion (97). OPA1 has also been discovered to be involved in mitochondrial crista remodeling with inner membrane organization (99).

Decreased mRNA levels of MFN2 have been found in term PE placentas (100). However, a proteomics analysis found the upregulated expression of MFN2 in pre-term early-onset PE placentas compared with pre-term controls (34). The gene expression of OPA increased 2.5-fold in pre-term early-onset PE placentas compared with term controls, but there was no difference between term early-onset PE and term late-onset PE placentas (59). In contrast, the protein expression of the l-OPA1/s-OPA1 ratio and OPA1 significantly increased in placenta from term PE patients compared with term controls, but the difference was not observed between pre-term PE placenta and pre-term normal placenta (101). Another study found decreased protein expression of OPA1 in pre-term PE placentas compared with pre-term controls (80). Neither MFN1/2 nor OPA1 was altered in term PE placentas compared with controls (27), but OPA1 was upregulated in the myometrium of pre-term early-onset PE compared to pre-term controls (58). OPA1 and MFN1/2 were downregulated in severe PE placentas (55). Although the findings of mitochondrial fusion-related genes in PE patients are inconsistent, the results in PE-like trophoblast cells are coincident. Decreased mRNA and protein levels of MFN2 have been confirmed in hypoxia-induced TEV-1 cells (100), and decreased transcript levels of MFN1 and MFN2 have been found in hypoxia-induced BeWo cells (27).

### Mitochondrial Fission

**Figure 6:**
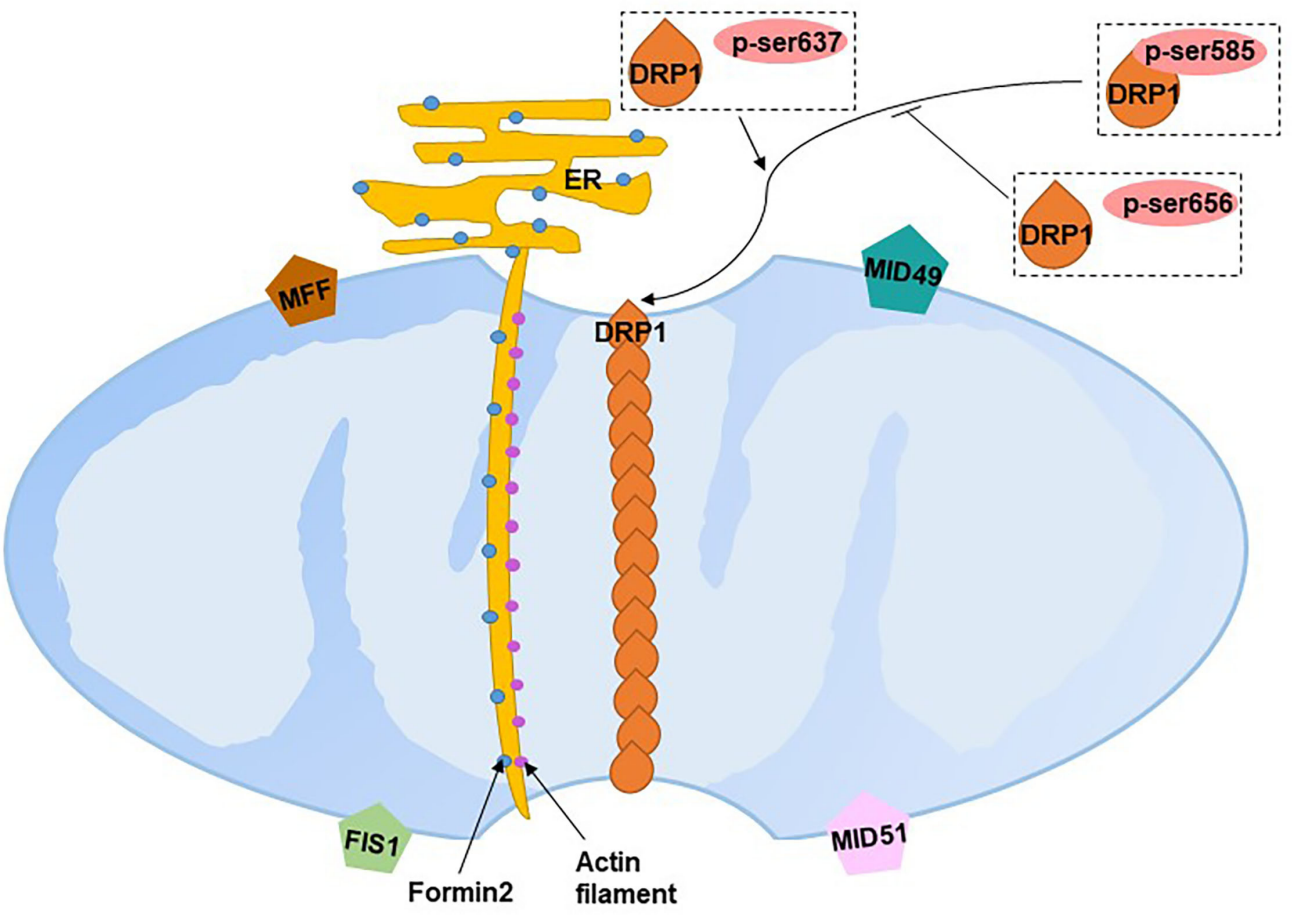
Mitochondrial fission. ER, endoplasmic reticulum; DRP1, dynamin-related protein 1; FIS1, fission 1; MFF, mitochondrial fission factor; MID49, mitochondrial dynamics protein of 49 kDa; MID51, mitochondrial dynamics protein of 51 kDa. Blue dots on the ER refer to formin 2, purple dots between the mitochondria and endoplasmic reticulum refer to actin filaments.

Fission segregates and uncouples damaged mitochondrial sub-organelles by dividing mitochondria from mitochondrial networks to maintain adequate numbers of mitochondria. Mitochondrial fission is primarily carried out by dynamin-related protein 1 (DRP1), a large GTPase. The translocation of DRP1 from the cytosol to mitochondria interacts with four receptor proteins in the OMM: fission 1 (FIS1), mitochondrial fission factor (MFF), mitochondrial dynamics protein of 49 kDa (MID49) and MID51, which initiate fission by constricting mitochondria (102). The translocation of DRP1 requires the phosphorylation-dephosphorylation at Ser of DRP1: Ser585 is phosphorylated by Cdk1/Cyclin B leading to the increased DRP1 GTPase activity at the onset of mitosis (103); the Ser656 phosphatased by PKA inhibits mitochondrial scission (104); and, dephosphorylation at Ser637 by the mitochondrial phosphatase phosphoglycerate mutase family member 5 recruits DRP1 to mitochondria to drive scission (105–107). However, DRP1 recruitment at the reticulum–mitochondria contact site has been found to occur prior to recruitment at the mitochondria constrict site, where the mitochondrion starts to segregate (108). The endoplasmic reticulum tubules first wrap around the constricted parts of mitochondria in the form of rings (108). Actin filaments then accumulate between mitochondrial and inverted formin 2-enriched endoplasmic reticulum membranes at the constriction sites, which initially recruits DRP1 to drive mitochondrial fission (109) (Figure 6). DRP1, FIS1 and MFF have been found to localize to peroxisomal membranes involved in peroxisomal fission (110).

A recent study found two distinct types of fission on African green monkey Cos-7 cells and mouse cardiomyocytes (111). Fission at the mitochondrial periphery (<25% from a tip) divides damaged mitochondria into smaller daughter mitochondria for sequent mitophagy, whereas division at the mid-zone of mitochondria (within the central 50%) leads to mitochondrial biogenesis (111). Compared with mid-zone fission, mitochondrial fission occurring at the periphery tends to have the following characteristics: reduced mitochondrial membrane potential, matrix pH, elevated ROS and increased Ca^+^. Although both types are regulated by DRP1, mitochondrial-endoplasmic reticulum contact, actin preconstruction and MFF play crucial roles in mid-zone fission, whereas mitochondrial-lysosomal contact and FIS1 play essential roles in peripheral fission. Divided mitochondrial fragmentation has two fates. Daughter mitochondria with higher membrane potential (presumably good quality mitochondria) proceed to fusion, while depolarized daughter mitochondria (presumably bad quality mitochondria) are degraded by mitophagy (112).

PE placentas show increased numbers of mitochondria in CTs, but with reduced size (80, 113), which is suggestive of increased mitochondrial fission. Although the mRNA transcript level of FIS-1 was not changed (55, 57), the fission-related dynamin-1-like protein (DNM1L) protein and mRNA transcript levels were increased in placentas complicated with PE (57). The protein expression of FIS1 was decreased in term PE relative to term controls (101). Increases in DRP1 expression, activation and phosphorylation have been found in pre-term PE placentas compared with pre-term controls (80). However, there was no difference in DRP1 expression levels in either pre-term PE or term PE compared with corresponding controls (55, 59). Neither FIS-1 nor DNM1L was unaltered in hypoxia-induced placental explants, but both were increased in hypoxia-induced BeWo cells (27).

### The Interaction Between Mitochondrial Dynamics and Biogenesis/Mitophagy

Many studies have found that mitochondrial biogenesis, mitophagy, fusion or fission could be simultaneously affected by external stimuli, but the interaction between mitochondrial dynamics and biogenesis/mitophagy has rarely been confirmed by intervention one molecule to observe the effect on another mQC molecule. Mitochondrial biogenesis has been found to be mediated by fusion- and fission-related proteins (114), but this lacks further confirmation. Mitochondrial dynamics are closely related to mitophagy. MFN2 phosphorylated by PINK1 regulates the recruitment of Parkin to depolarize mitochondria and facilitates Parkin-mediated ubiquitination (115). Chen and Dorn (115). found that the absence of MFN2 in mouse cardiac myocytes prevented the translocation of Parkin to depolarized mitochondria and then inhibited mitophagy OPA1 has also been shown to interact with lysine 70 of FUNDC1, which facilitates OPA1-mediated fusion, moreover, mutants of lysine 70 inhibit the interaction and thus promote FUNDC1-regulated mitophagy (116). Overexpressing OPA1 reduces the majority of mitophagy (117). Fission is required for mitophagy, as evidenced by mitophagy being prevented with a dominant-negative mutant of DRP1 (117, 118). Inhibition of mitochondrial fission by lowering the expression of FIS1 also reduces mitochondrial mitophagy (117, 118). Fusion and fission have been shown to be paired consecutive events, and fission quickly follows fusion (117). Furthermore, DRP1 interacts with MFN to increase elongated mitochondria by promoting fusion and inhibiting fission (119). However, the interaction between mitochondrial dynamics and biogenesis/mitophagy in PE has not been investigated, and this requires further studies to be helpful to explore the mQC-targeted treatment.

## Summary of Current Studies in Humans and Future Directions

Ten human studies have reported the alteration of mQC-related molecules in PE since 2015, while there are several limitations: (1) The numbers of clinical cases were small, ranging from 10 to 33. (2) 60% of these studies did not elucidate where the examined tissues were collected (e.g., at the maternal side or the fetal side). (3) In 30% of these studies, the gestational week at delivery between control and PE groups was not comparable. The expression of mQC-related molecules varies on the different gestational weeks, and thus the gestational week should be equivalent for comparison. (4) Only two studies isolated mitochondrial mRNA and protein from total mRNA and protein. The subcellular organelle where the mRNA or protein extracted is critical for molecular examination because several mQC-related molecules widely distribute in the cytosol, nucleus and mitochondrion, but the research scope of the above studies limit to the mitochondria. (5) The assessment of mQC-related molecules in humans should be verified in trophoblast cells or RUPP rats to reduce the bias of species differences and large fluctuation of human individuals. Therefore, future studies should be performed with larger sample size, comparable gestational weeks, clear distinction of the site where tissues were collected and the subcellular organelle where the molecules were assessed, and verification of results in other species. The future comprehensive human mQC-related PE studies will help to provide the clinical basis for mQC-targeted treatment of PE.

## Conclusion

mQC has been emerged as the treatment target of several diseases, including neurodegeneration, cardiovascular disease and cancer. Current studies have revealed that mQC-related molecules are associated with PE, although there are several drawbacks of these studies. These findings suggest that mQC plays an important role in the progression of PE, but further investigations for deeper elucidation are required. The future investigation of mQC in PE may provide a new insight on prevention and therapy strategies for PE.

## Author Contributions

XP contributed to relevant studies collection and summarisation and drafted manuscript. RH and YY: contributed to relevant studies collection and summarisation. ZL and YC: contributed to project conception and critical revision of manuscript. All authors contributed to the article and approved the submitted version.

## Funding

This work has been supported by National Natural Science Foundation of China (NSFC-82000399).

## Conflict of Interest

The authors declare that the research was conducted in the absence of any commercial or financial relationships that could be construed as a potential conflict of interest.

## Publisher's Note

All claims expressed in this article are solely those of the authors and do not necessarily represent those of their affiliated organizations, or those of the publisher, the editors and the reviewers. Any product that may be evaluated in this article, or claim that may be made by its manufacturer, is not guaranteed or endorsed by the publisher.
